# Measuring the casemix of physician practices in primary-care reform models in Ontario, Canada

**DOI:** 10.1186/1472-6963-11-S1-A22

**Published:** 2011-10-19

**Authors:** L Sibley, RH Glazier, B Hutchison

**Affiliations:** 1 Institute for Clinical Evaluative Sciences, Sunnybrook Health Sciences Centre, Toronto, Ontario, ON M4N 3M5, Canada; 2 Department of Family and Community Medicine, University of Toronto, Ontario, M5A 2N4, Canada; 3 Centre of Health Economics and Policy Analysis, McMaster University, Ontario, L8S 4L8, Canada; 4 Department of Family Medicine, McMaster University, Ontario, L8S 4L8, Canada

## Introduction

A number of different blended-payment models for primary-care delivery have been introduced in Ontario, Canada over the last decade. These models have different incentives and, therefore, have attracted different physicians and patients, depending upon their geographical location and practice characteristics.

As policy makers at the Ministry of Health and Long-Term Care evaluate and consider possible changes to these models, it is important that they be able to characterize the Casemix of patients who are enrolled to them, and to understand the healthcare needs of those who have not enrolled with a primary-care model. This study evaluates a method for summarizing the Casemix of primary-care rosters, and it examines the variations of Casemix between and within the different model types.

## Methods

The study population includes all residents of Ontario who were registered with the Ontario Health Insurance Plan (OHIP) on March 31, 2010, and all primary-care physicians who belonged to a primary-care reform model on the same date. Each individual in the province was assigned a morbidity weight using the Johns Hopkins Adjusted Clinical Groups (ACG) Casemix System along with diagnosis data collected during the previous year. The Casemix of each physician’s roster was summarized with a Standardized ACG Morbidity Index (SMI), which is the standardized average morbidity weight of all patients on the roster. The roster SMIs were compared across and within the three following group types: enhanced fee-for-service, capitation, and team-based capitation.

## Results

The study sample included 6,579 physician rosters which consisted of 9,225,428 patients. The mean SMI of enhanced fee-for-service rosters was higher than the SMI for both types of capitation groups (1.22 vs. 1.03; p<0.001). The interquartile range of the enhanced fee-for-service rosters (1.30-0.93) was greater than both the capitation rosters (1.20-0.88) and the team-based capitation rosters (1.18-0.88). The 95th percentile of the enhanced fee-for-service rosters was 1.74 with the other two groups having a 95th percentile of 1.52.

## Conclusions

The rosters of physicians in enhanced fee-for-service groups have a higher average morbidity burden and greater variation in morbidity than the capitation group rosters. Being able to easily and reliably measure the morbidity burden allows decision makers to identify and fairly reimburse physicians whose patients have a higher burden of illness.

